# ADHD SYMPTOMOLOGY AND COGNITIVE PERFORMANCE IN LATER LIFE

**DOI:** 10.1093/geroni/igae098.3283

**Published:** 2024-12-31

**Authors:** Marrium Mansoor, Ben Katz

**Affiliations:** Virginia Tech, Blacksburg, Virginia, United States; Virginia Tech, Blacksburg, Virginia, United States

## Abstract

Attention Deficit Hyperactivity Disorder (ADHD) is a neurodevelopmental disorder affecting up to 6.7% of adults worldwide. Validated questionnaires to diagnose ADHD often include scales for both the inattention and hyperactivity symptoms which characterize this disorder. Although inattention symptoms have been previously linked to cognitive performance in younger samples, very few studies have examined links between these ADHD symptoms and cognitive performance in adult samples, and very few have done so with middle aged and older adults. In this study, we drew from a nationally representative sample from the Health and Retirement Study (HRS) of ~ 1400 middle to older adults (M = 69 years, SD = 10.3; Range = 55-107 years) who completed a set of cognitive measures and an ADHD symptomology questionnaire. We found statistically significant associations between self-reported symptoms of inattention and cognitive measures including Number Series (B=-0.056, p=0.036), Serial 7s (B=-0.088, p< 0.001), Delayed Recall (B=-0.112, p< 0.001), Immediate Recall (B=-0.116, p< 0.001), and global cognition (B=-0.148, p< 0.001) while controlling for age, gender, years of education, and self-rated health. Hyperactivity symptoms were only significantly predictive of global cognition (B=-0.077, p=0.01) and no other measures. These results are consistent with previous work on the links between ADHD symptomology and cognitive performance in younger populations and add to the literature on ADHD in later life. This may have implications for clinicians and practitioners as well as future research on older adults with ADHD.

